# Kavain, the Major Constituent of the Anxiolytic Kava Extract, Potentiates GABA_A_ Receptors: Functional Characteristics and Molecular Mechanism

**DOI:** 10.1371/journal.pone.0157700

**Published:** 2016-06-22

**Authors:** Han Chow Chua, Emilie T. H. Christensen, Kirsten Hoestgaard-Jensen, Leonny Y. Hartiadi, Iqbal Ramzan, Anders A. Jensen, Nathan L. Absalom, Mary Chebib

**Affiliations:** 1 Faculty of Pharmacy, The University of Sydney, Sydney, New South Wales, Australia; 2 Department of Drug Design and Pharmacology, Faculty of Health and Medical Sciences, University of Copenhagen, Copenhagen, Denmark; Dalhousie University, CANADA

## Abstract

Extracts of the pepper plant kava (*Piper methysticum*) are effective in alleviating anxiety in clinical trials. Despite the long-standing therapeutic interest in kava, the molecular target(s) of the pharmacologically active constituents, kavalactones have not been established. γ-Aminobutyric acid type A receptors (GABA_A_Rs) are assumed to be the *in vivo* molecular target of kavalactones based on data from binding assays, but evidence in support of a direct interaction between kavalactones and GABA_A_Rs is scarce and equivocal. In this study, we characterised the functional properties of the major anxiolytic kavalactone, kavain at human recombinant α1β2, β2γ2L, αxβ2γ2L (x = 1, 2, 3 and 5), α1βxγ2L (x = 1, 2 and 3) and α4β2δ GABA_A_Rs expressed in *Xenopus* oocytes using the two-electrode voltage clamp technique. We found that kavain positively modulated all receptors regardless of the subunit composition, but the degree of enhancement was greater at α4β2δ than at α1β2γ2L GABA_A_Rs. The modulatory effect of kavain was unaffected by flumazenil, indicating that kavain did not enhance GABA_A_Rs via the classical benzodiazepine binding site. The β3N265M point mutation which has been previously shown to profoundly decrease anaesthetic sensitivity, also diminished kavain-mediated potentiation. To our knowledge, this study is the first report of the functional characteristics of a single kavalactone at distinct GABA_A_R subtypes, and presents the first experimental evidence in support of a direct interaction between a kavalactone and GABA_A_Rs.

## Introduction

The use of intoxicating substances to enhance mood, alter consciousness and to achieve spiritual enlightenment is known in virtually every culture. Alcohol is the most commonly used intoxicant globally, with the exception in a few regions [1]. For millennia, Pacific Islanders have been using the root of a native pepper plant called kava (*Piper methysticum*) to prepare a non-alcoholic psychoactive beverage, which is also called kava. Kava drinking is an integral component of the Pacific Islander culture, with kava playing roles as a sacred drug in religious rituals, a social lubricant at formal gatherings, and a medicine to induce relaxation and sleep [2]. The contemporary use of kava extends beyond ritualised circumstances [3, 4]. In some western societies, kava is used as a prescription-free alternative to the benzodiazepines to relieve stress-induced anxiety and insomnia [5]. The recreational use of kava as a substitute for alcohol is also gaining popularity owing to kava’s calming effect which contrasts the aggressive tendencies associated with alcohol [6].

There is a long-standing interest in the use of kava in the treatment of anxiety. In clinical trials, kava extracts are superior to placebo in reducing anxiety, and are generally well tolerated with negligible to mild side effects [7–9]. Despite reports of alleged kava-induced hepatotoxicity which led to the withdrawal and restriction of kava in several countries [10, 11], systematic reviews and meta-analyses conducted over the last 15 years found a clear positive benefit-to-risk ratio for kava [12, 13]. In view of the lack of direct causal evidence for liver injury, the ban of kava in Germany was recently overturned, leading to a resurgence of attention on kava [14]. Currently, there is an ongoing phase III clinical trial aimed to establish the efficacy and safety of kava in patients diagnosed with generalised anxiety disorder [15].

**Fig 1:**
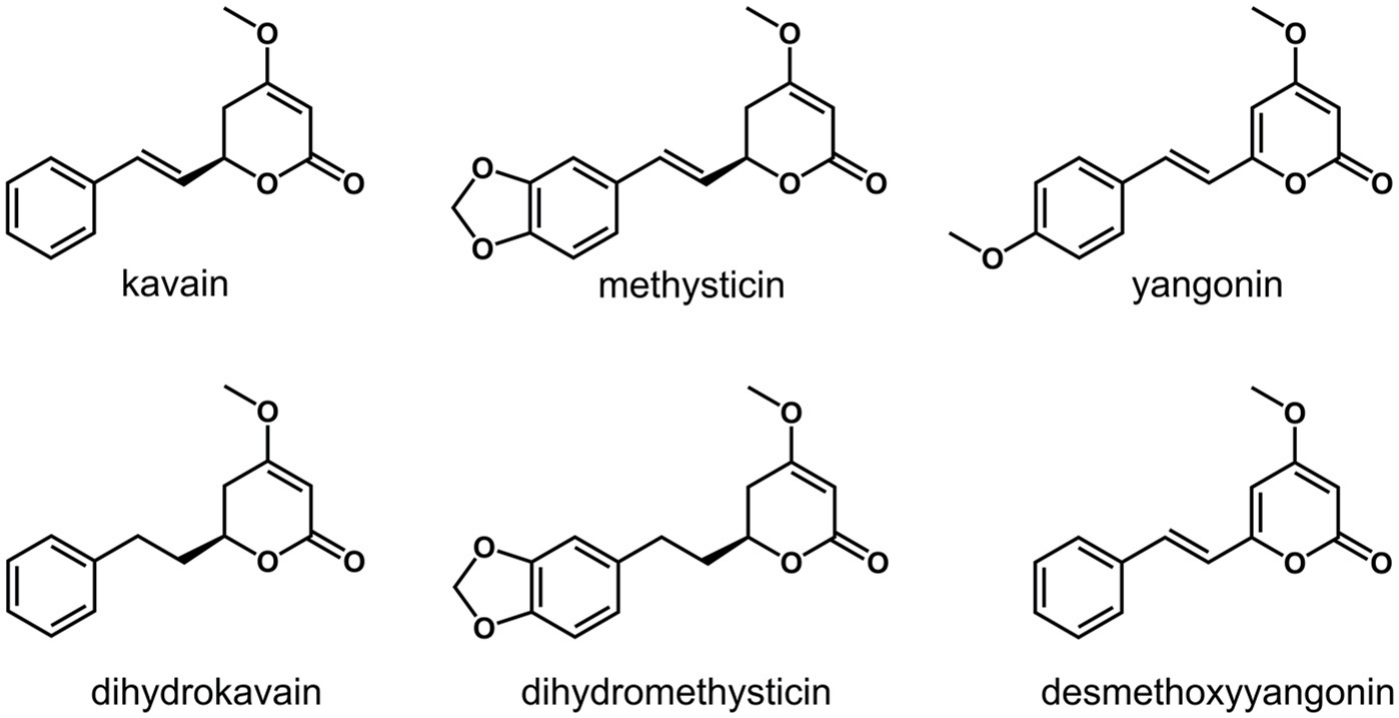
Chemical structures of the six major kavalactones found in kava.

A group of structurally-related, lipophilic compounds known as kavalactones (or kavapyrones) is responsible for the clinical effects of kava [16]. Kavain, along with dihydrokavain, methysticin, dihydromethysticin, yangonin, and desmethoxyyangonin are the most abundant kavalactones (Fig 1) [17]. Numerous proteins including γ-aminobutyric acid type A receptors (GABA_A_Rs), voltage-gated Na^+^ and Ca^2+^ channels, opioid μ and δ receptors, dopamine type-2 receptor, histamine type-1 and 2 receptors, cannabinoid type-1 receptor, and monoamine oxidase type B have been suggested to be the molecular targets for kavalactones [16, 18–20]. Due to the paucity of robust evidence, however, a consensus on the pharmacology of kavalactones has not yet been reached, but there is a prevailing view on the basis of their benzodiazepine-like pharmacological actions that GABA_A_Rs are the main target for kavalactones.

GABA_A_Rs are a class of physiologically and therapeutically important ligand-gated ion channels (LGICs). These pentameric receptors occur in the brain with diverse composition that arise from a large number of subunits (α1–6, β1–3, γ1–3, δ, ε, θ and π). This structural heterogeneity confers highly complex pharmacology to GABA_A_Rs. An impressive range of clinically used therapeutics such as benzodiazepines, barbiturates and anaesthetics are known to bind to distinct allosteric sites found on GABA_A_Rs to modulate receptor function. To date, evidence suggestive of an interaction between kavalactones and GABA_A_Rs mainly comes from radioligand binding studies. In rodent brain membranes, kava extracts with enriched kavalactone content and pure kavalactones have been shown to enhance binding of orthosteric radioligands to native GABA_A_Rs [19, 21–23]. Notably, none of these studies detected significant affinity of kavalactones for the benzodiazepine binding site, contrary to popular belief.

Despite the findings of these studies, a direct interaction between kavalactones and GABA_A_Rs has not been conclusively established due to several reasons. First, the contribution of non-kavalactone compounds in enhancing ligand binding cannot be ruled out with the use of kava extracts in some studies. Second, kavalactones were tested on a heterogeneous population of GABA_A_Rs isolated from rodent brain, thus the identity of specific GABA_A_R subtypes modulated by kavalactones is not known. Third, due to the weak affinity of kavalactones and the lipid-dependent activity reported by Davies *et al*. (1992) [22], the possibility for these lipophilic compounds to modulate GABA_A_Rs indirectly by altering the physicochemical properties of lipid membrane cannot be ruled out.

To elucidate the molecular basis of the interaction between kavalactones and GABA_A_Rs, we performed functional characterisation of the kavalactone kavain at 9 human GABA_A_R subtypes expressed in *Xenopus* oocytes. Kavain was selected for these studies due to its abundance in kava extracts commonly consumed [24], as well as its established anxiolytic and sedative properties in animals and humans [25–27]. We demonstrate that the modulatory effect of kavain at GABA_A_Rs occurs in a subtype non-selective and flumazenil-insensitive manner. We also present evidence, for the first time, that kavain action at GABA_A_Rs is attenuated by the anaesthetic-impairing β3N265M point mutation.

## Materials and Methods

### Chemicals

GABA, DL-kavain, flumazenil, propofol, zinc chloride (ZnCl_2_), DMSO and all buffer ingredients mentioned in this study were purchased from Sigma-Aldrich (St. Louis, MO, USA); diazepam was purchased from Apin Chemicals LTD (Abingdon, Oxon, UK); allopregnanolone and DS2 (4-chloro-N-(2-thiophen-2-ylimidazo[1,2-a]pyridin-3-yl)benzamide) were purchased from Tocris Bioscience (Bristol, UK). Etomidate hydrochloride (HCl) was synthesised following protocols previously described in Janssen *et al*. [28] and Janssen *et al*. [29].

### Molecular biology

Human complementary DNAs (cDNAs) of GABA_A_R subunits used in this study and their corresponding subcloned vectors include: α1, β1, β2 and γ2L in pCDM8; α2 and α3 in pCDNA3.1; α4 and δ in pCDNA1/Amp; α5 in pCDNA3; β3 in pGEMHE. The identity of the subunits was confirmed with DNA sequencing (Australian Genome Research Facility, Westmead, NSW, Australia). Linearised plasmid DNAs were used as templates for the synthesis of messenger RNAs (mRNAs) using the mMessage mMachine T7 transcript kit from Ambion (Austin, TX, USA). Mutations were introduced to various GABA_A_R subunits of interest using the QuikChange II Site-Directed Mutagenesis Kit. For the α1M236W point mutation, forward primer 5′-CCTGCCGTGCATATGGACAGTTATTCTC-3′ and reverse primer 5′-GAGAATAACTGTCCATATGCACGGCAGG-3′ were used. For the β2M286W point mutation, forward primer 5′-AGGCCATTGACATGTACCTGTGGGGGTGCTTTGTCTTCGT-3′ and reverse primer 5′- ACGAAGACAAAGCACCCCCACAGGTACATGTCAATGGCCT-3′ were used. For the β3N265M point mutation, forward primer 5′- ACAATGACAACCATCATGACCCACCTTCGGGAG-3′ and reverse primer 5′-CTCCCGAAGGTGGGTCATGATGGTTGTCATTGT-3′ were used.

### *Xenopus* oocytes isolation and preparation

All the procedures involved in the use of *Xenopus laevis* frogs were approved by the Animal Ethics Committee of the University of Sydney (Reference number: 2013/5915). *Xenopus laevis* frogs were housed in tanks containing compatible triplets on a 12-hour light/dark cycle in a reticulated aquatic system of 15 cm water depth. The aquatic solution was maintained at 18–22°C and persistently filtered with 50 μm carbon filters and routinely monitored for pH, nitrate levels and water hardness. The frogs were fed Wardley’s cichlid floating pellets (Hartz Mountain Corporation NJ, USA) twice weekly and Wardley’s reptile sticks with fortified calcium at least weekly. Tanks were cleaned twice weekly on the day after feeding to reduce nitrate levels. Mature female *Xenopus laevis* frogs were anaesthetised with 0.17% tricaine (buffered with 0.06% sodium bicarbonate) for 15 minutes, after which the loss of righting reflex was confirmed before transferring on to ice where surgeries were performed. A small (1–2 cm) abdominal incision was made through both the skin and muscle layer with surgical knives. Ovary lobes were removed with a pair of forceps, and kept in oocyte releasing 2 (OR2) buffer (82.5 mM NaCl, 2 mM KCl, 1 mM MgCl_2_, 5 mM HEPES hemisodium; pH 7.4). The skin and muscle layer were sutured separately, and frogs were allowed to recover for six months before they were reselected for surgeries. A total of five recoverable surgeries were allowed on each frog, before a terminal surgery was performed, in which a lethal dose of tricaine (0.5%) was used. Removed ovary lobes were divided into small sections and treated with 2 mg/mL Collagenase A (Boehringer Mannheim, Indianapolis, IN, USA) in OR2 buffer for at least an hour to defolliculate the oocytes. The released oocytes were then rinsed in ND96 storage solution (96 mM NaCl, 2 mM KCl, 1 mM MgCl_2_, 1.8 mM CaCl_2_, 5 mM HEPES hemisodium, 2.5 mM pyruvate, 0.5 mM theophylline; pH 7.4). Healthy-looking stage V–VI oocytes were isolated and kept in ND96 storage solution at 18°C until ready for injection.

### Electrophysiological recording of recombinant GABA_A_Rs in *Xenopus* oocytes

The expression and functional characterisation of recombinant GABA_A_Rs in *Xenopus* oocytes were performed as previously described [30]. The subunit mRNA ratios for the expression of αβγ receptors were adjusted according to the β subunit isoform in that when β2-containing receptors were expressed, a 1:1:3 ratio was used, whereas when β1- or β3-containing receptors were expressed, a 10:1:10 ratio was used to minimise the expression of homomeric β1/3 homomeric receptors. To express α4β2δ receptors, a 5:1:5 ratio was used, and the pharmacological characteristics of this receptor were as described in Hartiadi *et al*. (2016) [31]. The injection ratio used for the expression of α1β2 and β2γ2L receptors were 1:1 and 1:3 respectively. Approximately 2 to 10 ng of mRNA mixture was injected into each oocyte. Injected oocytes were stored in ND96 storage solution supplemented with 50 μg/mL gentamycin and tetracycline at 18°C.

Two-electrode voltage clamp recordings were performed on oocytes 2–5 days post-injection at room temperature (20–25°C) using a GeneClamp 500B amplifier (ADInstruments, Sydney, NSW, Australia) or a Warner OC-725C Oocyte Clamp (Warner Instrument Corp, Hamden, CT, USA). All experiments were conducted at a holding potential of –60 mV, and data were acquired with a PowerLab 2/25, and LabChart (version 5.0.2) software (ADInstruments, Sydney, NSW, Australia). The recording microelectrodes (0.2–1.1 MΩ) were generated from capillary glass (Harvard Apparatus, Holliston, MA, USA) using a single-stage glass microelectrode puller (Narishige, Tokyo, Japan), and were filled with 3 M KCl solution. The oocyte was continuously perfused with ND96 recording solution (96 mM NaCl, 2 mM KCl, 1 mM MgCl_2_, 1.8 mM CaCl_2_, 5 mM HEPES hemisodium; pH 7.4) at an approximate rate of 5 mL/min.

GABA, etomidate and ZnCl_2_ were dissolved in ND96 recording solution, whereas allopregnanolone, diazepam, DS2, flumazenil and propofol were dissolved in DMSO to make up stock solutions of desired concentrations. GABA stock solutions were prepared fresh on the day of experiments. When working with chemicals dissolved in DMSO, all perfusates were standardised to contain 0.8% DMSO, which did not produce any alteration in the recordings. Perfusates containing these chemicals were made up fresh before each application. Depending on the concentration and the solubility of the chemicals applied on oocytes, the wash-out periods ranged from 3 to 15 minutes to allow time for receptors to recover from desensitisation. All experiments were performed using at least two different batches of oocytes.

### Data analysis

Prism (version 5.04; GraphPad Software, La Jolla, CA, USA) was used for data analysis. All data are presented as mean ± standard error of the means (SEM). Raw data from GABA concentration-response experiments were fitted to a monophasic Hill equation, and the fitted maximal values were used for normalisation of each dataset. In this equation, *I*_max_ represents the maximal agonist response, [*A*] represents the agonist concentration, EC_50_ represents the agonist concentration required to activate 50% of the maximal response, *n*_H_ represents the Hill slope of the fitted curve.

$$I=\frac{I_{\text{max}}}{1+10^{{}^{\left((\text{logE}{\text{C}}_{50}{}_{}-[A])n_{\text{H}}\right)}}}$$ (1)

To illustrate the modulatory effect of a chemical of interest, the data were normalised and expressed as shown in Eq 2, where *I* represents the current responses elicited by the co-application of GABA and modulator, *I*_*GABA*_ represents the amplitude of the control GABA-elicited responses. Alternatively, the modulatory effect was expressed as the fold of potentiation, as shown in Eq 3.

$$\frac{I-I_{\text{GABA}}}{I_{\text{GABA}}}$$ (2)

$$\frac{I}{I_{\text{GABA}}}$$ (3)

When comparing parameters across different groups (receptors subtypes/wild type *vs*. mutant), the means of the values obtained from individual dataset were analysed using either unpaired Student’s *t* test (comparing means for two groups) or one-way ANOVA with Tukey’s *post hoc* test (comparing every mean with every other mean in three or more groups). When comparing the effect before and after the addition of a drug, paired Student’s *t* test was used. Statistical significance was attained at *p* < 0.05.

## Results

### Functional characterisation of kavain at GABA_A_Rs

**Fig 2:**
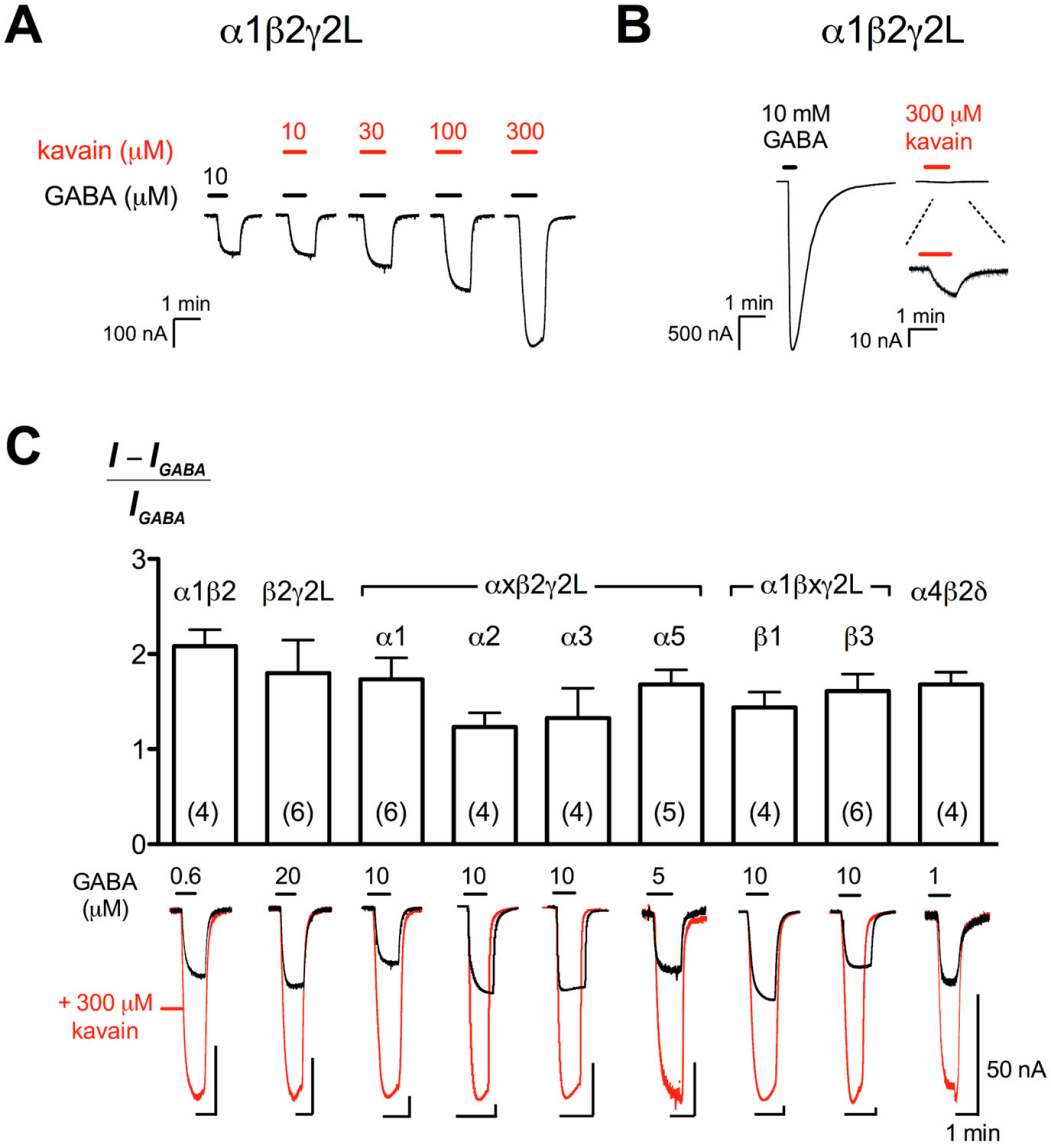
Kavain potentiates GABA_A_Rs with no apparent subtype selectivity. (A) Representative traces demonstrating kavain (10–300 μM) enhancing current elicited by 10 μM GABA (EC_3_) at α1β2γ2L GABA_A_Rs in a concentration-dependent manner. (B) Representative traces of current responses elicited by a maximal concentration of GABA (10 mM), in comparison to 300 μM kavain alone. (C) *Top panel*, Potentiation of GABA-elicited currents (EC_3-7_) at α1β2, β2γ2L, αxβ2γ2L (x = 1, 2, 3 and 5) and α1βxγ2L (x = 1, 2 and 3) GABA_A_Rs by 300 μM kavain. At α4β2δ GABA_A_Rs, the GABA control (1 μM) corresponds to an EC_30_. Data are presented as mean ± SEM. Numbers in bars indicate the number of experiments. No significant difference was found for kavain potentiation at these receptor subtypes (Tukey’s test; *p* > 0.05 for all pairwise comparisons). *Bottom panel*, Superimposed current traces of GABA alone (black) and GABA in combination with 300 μM kavain (red) at the corresponding receptor subtypes. The black bars above the traces indicate duration of drug application.

We first investigated the effect of kavain (10–300 μM) at α1β2γ2L GABA_A_Rs, the most abundant native GABA_A_R subtype, in the presence of GABA EC_3_ (10 μM), and found that kavain enhanced GABA-elicited responses in a concentration-dependent manner (Fig 2). The modulatory effect of kavain was moderate, with only 170 ± 23% of enhancement measured at 300 μM (*n* = 6; Fig 2C). We also investigated the ability of kavain to activate the receptors, and found negligible intrinsic agonist activity, with 300 μM kavain eliciting currents < 1% of the 10 mM GABA responses (*n* = 5; Fig 2B). To understand the subunit requirement for kavain modulation, we tested kavain (10–300 μM) at different GABA_A_R subtypes with a low concentration of GABA (EC_3-7_ for all receptors except α4β2δ, where the EC_30_ was used due to the low level of expression). The receptor subtypes investigated were α1β2, β2γ2L, αxβ2γ2L (x = 1, 2, 3 or 5), α1βxγ2L (x = 1, 2 or 3) and α4β2δ (GABA concentration-response relationships summarised in Table 1). Kavain (300 μM) potentiated current responses elicited by low concentrations of GABA at all receptors to a similar degree, exhibiting no significant subtype selectivity (no difference was detected for all pairwise comparisons using Tukey’s test; *p* > 0.5; Fig 2C).

**Table 1:** GABA concentration-response curve parameters at various receptor subtypes derived from curve-fitting procedures.

Receptor subtypes	GABA EC_50_ (95% CI)	*n*_*H*_ ± SEM	*n*
**α1β2**	7.8 (6.9–8.8) μM	1.2 ± 0.072	5
**β2γ2L**	1.3 (0.9–1.8) mM	0.63 ± 0.034	4
**α1β2γ2L**	136 (116–158) μM	0.99 ± 0.062	10
**α1β2γ2L (+ 300 μM kavain)**	99 (87–113) μM^a^	0.75 ± 0.026	6
**α2β2γ2L**	76 (72–80) μM	1.4 ± 0.041	4
**α3β2γ2L**	150 (136–162) μM	1.2 ± 0.054	4
**α5β2γ2L**	67 (56–80) μM	1.0 ± 0.078	5
**α1β1γ2L**	89 (79–100) μM	1.1 ± 0.059	6
**α1β3γ2L**	93 (83–104) μM	1.2 ± 0.070	5
**α4β2δ**	2.6 (1.7–4.1) μM	0.70 ± 0.095	4
**α4β2δ (+ 300 μM kavain)**	1.7 (0.93–3.1) μM^a^	0.59 ± 0.11	4
**α1M286Wβ2γ2L**	59 (48–73) μM**^b^	0.80 ± 0.053	7
**α1β2M286Wγ2L**	25 (23–27) μM****^b^	1.1 ± 0.036	5
**α1β3N265Mγ2L**	72 (65–80) μM^c^	1.4 ± 0.083	5

^*a*^ The GABA potency was not significantly altered in the presence of 300 μM kavain (*p* > 0.05 for logEC_50_ comparisons; paired *t* test).

^*b*^ The GABA potency was significantly enhanced compared to wild-type α1β2γ2L GABA_A_Rs (** *p* < 0.01; **** *p* < 0.0001 for logEC_50_ comparisons; unpaired *t* test).

^*c*^ The GABA potency was not significantly different from the wild-type α1β3γ2L GABA_A_Rs (*p* > 0.05 for logEC_50_ comparisons; unpaired *t* test).

### The differential effects of kavain at α1β2γ2L and α4β2δ GABA_A_Rs

**Fig 3:**
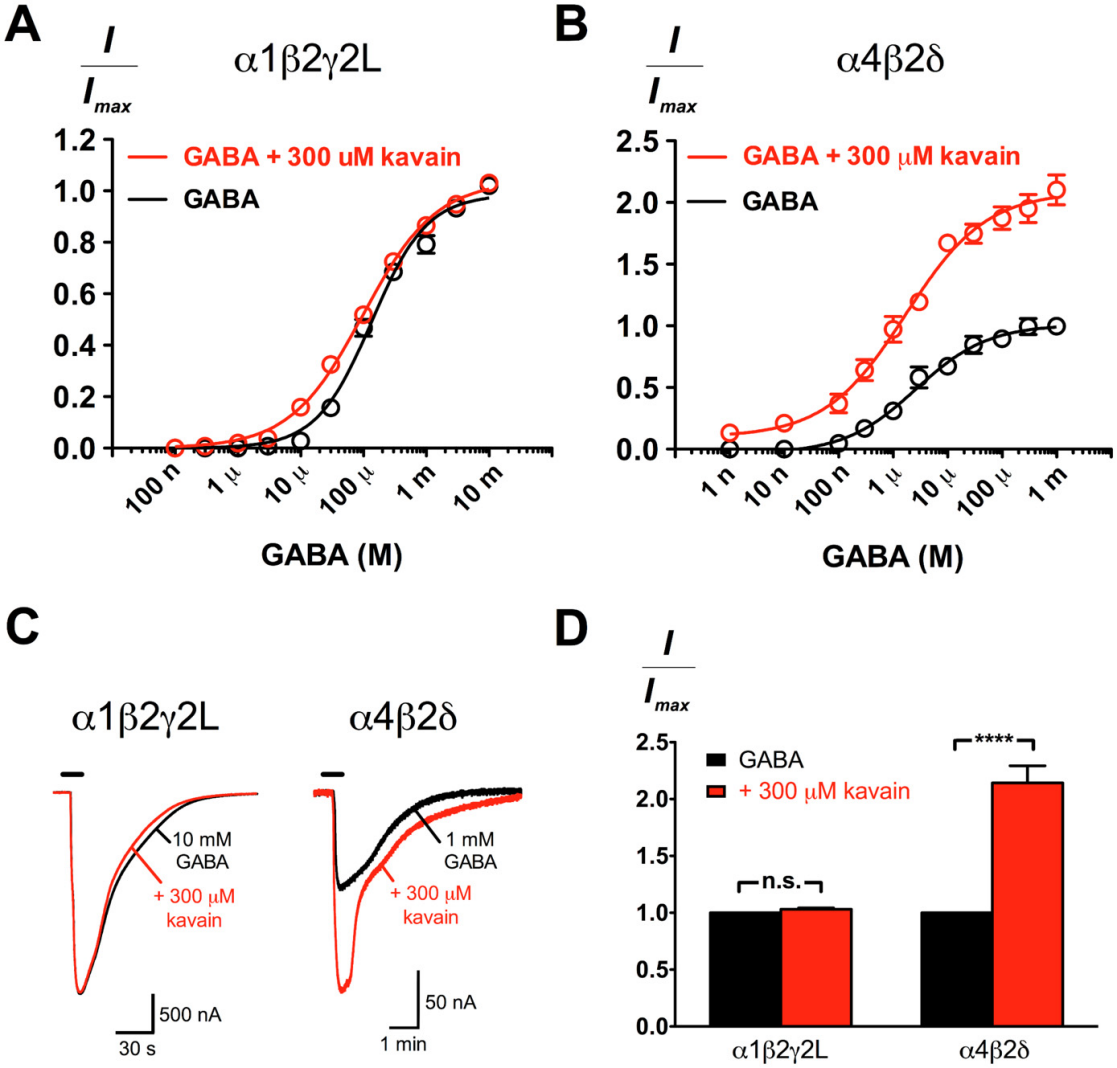
Kavain produced greater enhancement of GABA-elicited currents at α4β2δ than at α1β2γ2L GABA_A_Rs. (A) GABA concentration-response curves in the absence (black; *n* = 10) and presence (red; *n* = 6) of 300 μM kavain at α1β2γ2L GABA_A_Rs. The curve parameters are summarised in Table 1. (B) GABA concentration-response curves in the absence (black; *n* = 4) and presence (red; *n* = 4) of 300 μM kavain at α4β2δ GABA_A_Rs. The curve parameters are summarised in Table 1. (C) Superimposed current responses of maximal GABA in the absence (black) and presence (red) of 300 μM kavain at α1β2γ2L and α4β2δ GABA_A_Rs. (D) The effect of kavain on the maximal GABA current responses at α1β2γ2L (*n* = 6) and α4β2δ (*n* = 7) GABA_A_Rs was compared using the paired *t* test, and the significance levels are indicated with n.s. (not significant) and **** (*p* < 0.0001). Data are presented as mean ± SEM.

To understand the nature of kavain modulation in response to different GABA concentrations, we constructed GABA concentration-response curves in the absence and presence of 300 μM kavain at α1β2γ2L and α4β2δ receptors. These receptors represent the major synaptic and extrasynaptic GABA_A_Rs respectively, and are characterised by distinct subcellular localisations and GABA sensitivities (α1β2γ2L: EC_50_ = 136 μM; α4β2δ: EC_50_ = 2.6 μM; Table 1). At α1β2γ2L receptors, kavain showed modest enhancement at GABA concentrations below EC_45_, but did not affect the EC_50_ and maximal efficacy of GABA (*p* > 0.05 for both parameters; paired *t* test; Fig 3A and 3D; Table 1). In contrast, kavain increased the maximal response evoked by GABA at α4β2δ receptors by two fold (*p* < 0.0001; paired *t* test) without affecting the GABA potency significantly (*p* > 0.05 for logEC_50_ comparison; paired *t* test; Fig 3B and 3D; Table 1).

### Interaction of kavain with benzodiazepines and anaesthetics

The psychoactive properties of kavalactones have sparked concern of potential adverse pharmacodynamic interactions with the co-administration of kava with other CNS depressants, but there is a lack of experimental evidence to support these postulations [32]. Thus, we sought to determine whether kavain interacts with clinically used benzodiazepines and anaesthetic agents in an additive, synergistic or antagonistic manner.

#### Benzodiazepines

**Fig 4:**
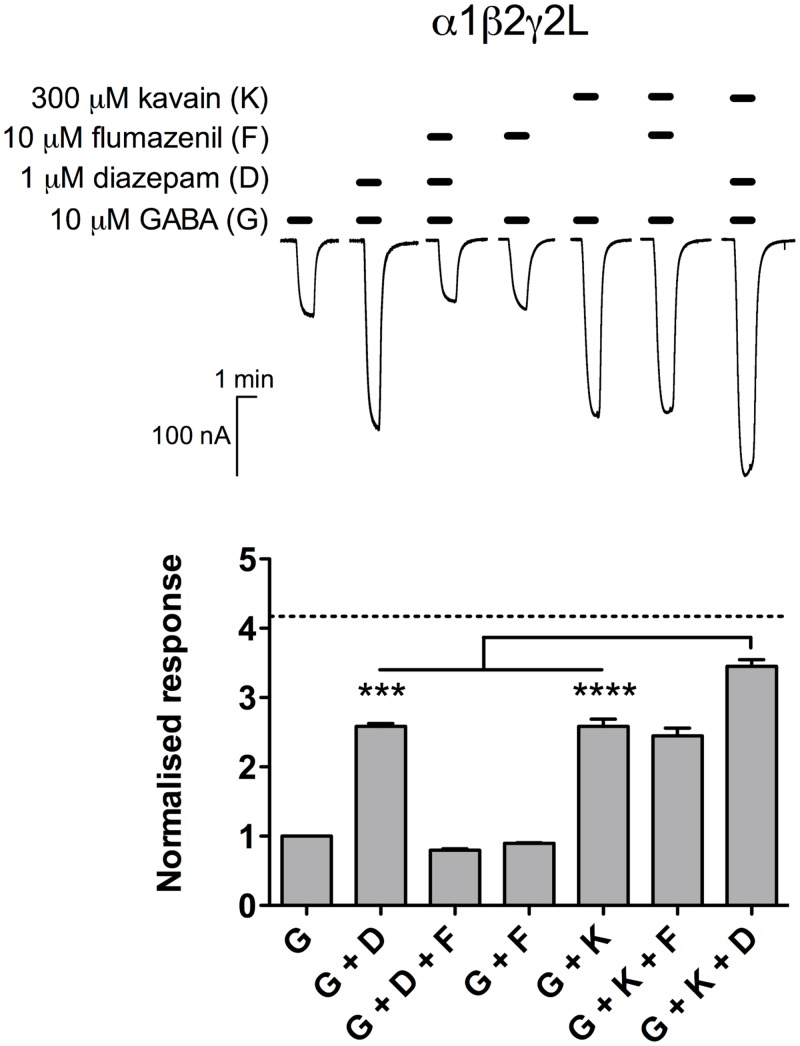
Flumazenil-insensitive kavain potentiation has less-than-additive effect on diazepam action. *Top*, Representative traces demonstrating responses to control (10 μM GABA); control and 1 μM diazepam; control, 1 μM diazepam and 10 μM flumazenil; control and 10 μM flumazenil; control and 300 μM kavain; control, 300 μM kavain and 10 μM flumazenil; and control, 300 μM kavain and 1 μM diazepam. *Bottom*, The modulatory effect of diazepam, flumazenil and kavain at α1β2γ2L GABA_A_Rs (*n* = 5). Kavain potentiation was unchanged in the presence of flumazenil (G + K *vs*. G + K + F; *p* > 0.05; paired *t* test). The combination of kavain and diazepam (G + K + D) resulted in greater potentiation than diazepam (G + D; *p* < 0.001; paired *t* test) and kavain (G + K; *p* < 0.0001; paired *t* test) alone, but the effect was less than the expected additive modulatory effect (dotted line). Data are normalised to the current responses elicited by 10 μM GABA, and are presented as mean ± SEM.

We investigated the effect of combining benzodiazepine site ligands with kavain at α1β2γ2L GABA_A_Rs (*n* = 5; Fig 4). In concordance with the literature, diazepam (1 μM) enhanced current responses elicited by 10 μM GABA in a flumazenil-sensitive manner, and flumazenil alone did not alter GABA responses, consistent with its neutralising modulator profile [33]. Unlike diazepam, the GABA-enhancing action of 300 μM kavain was unaffected in the presence of flumazenil. We also found that the co-application of kavain and diazepam resulted in significantly greater enhancement of GABA current (350 ± 10%) compared to their actions alone (GABA + diazepam: 260 ± 4.2%, *p* < 0.001; GABA + kavain: 260 ± 11%, *p* < 0.0001; paired *t* test; Fig 4B). However, this degree of potentiation was less than additive (expected additive value = 420%). The same experiment was repeated with 100 nM diazepam, and a less-than-additive interaction was detected again with the kava and diazepam combination (*n* = 3; data not shown).

#### General anaesthetics

The effect of combining kavain and general anaesthetics (etomidate and propofol) on receptor modulation was also investigated at α1β2γ2L GABA_A_Rs. A low and a high concentration of the general anaesthetics were used (3 and 30 μM for etomidate; 10 and 100 μM for propofol) to produce the modulatory and agonist effects respectively.

#### Etomidate

**Fig 5:**
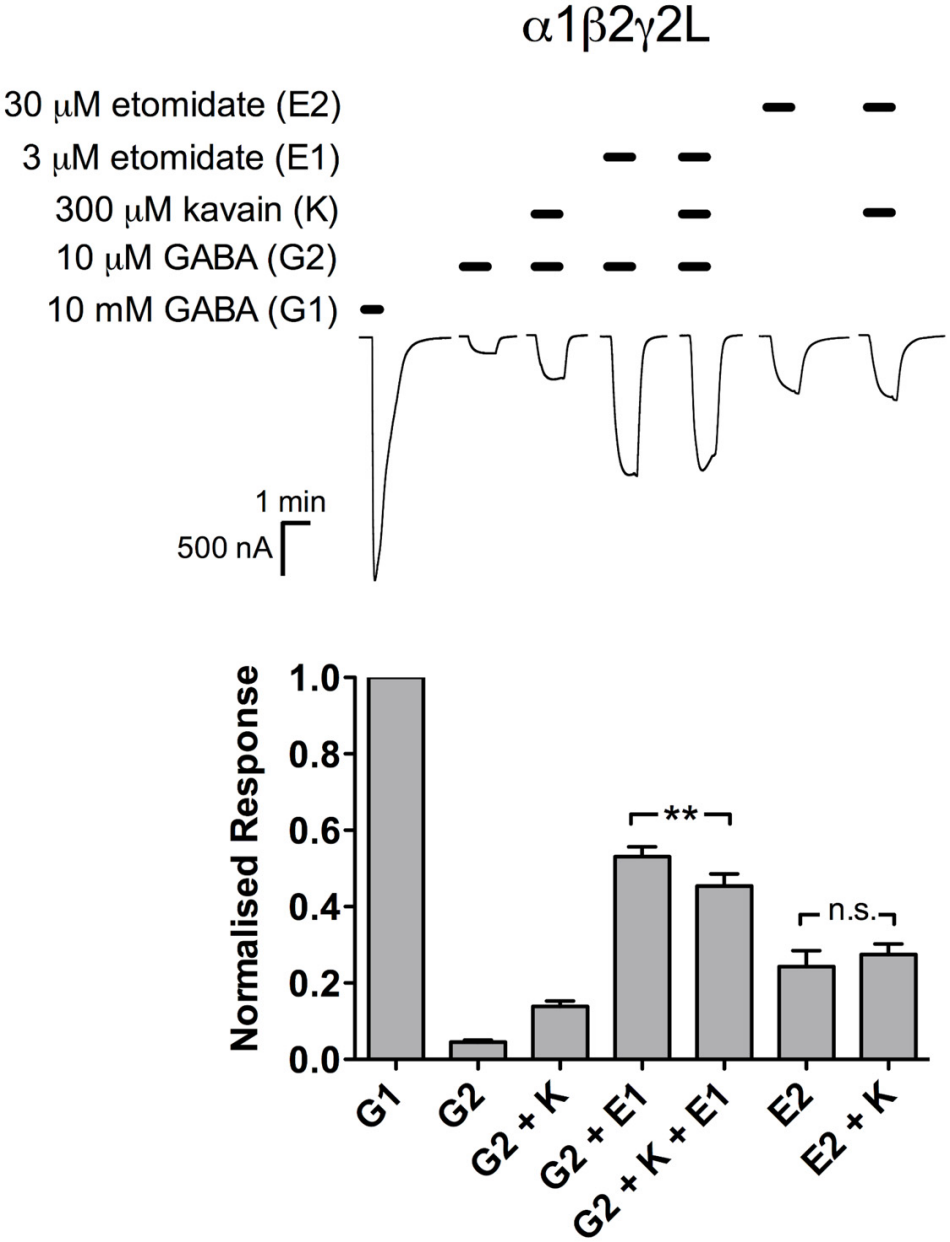
Kavain modestly reduced etomidate potentiation, but did not affect the direct activation caused by etomidate at α1β2γ2L GABA_A_Rs. *Top*, Representative traces of current responses elicited by 10 mM GABA (control); 10 μM GABA; 10 μM GABA and 300 μM kavain; 10 μM GABA and 3 μM etomidate; 10 μM GABA, 300 μM kavain and 3 μM etomidate; 30 μM etomidate; 30 μM etomidate and 300 μM kavain. *Bottom*, Kavain caused a subtle but significant reduction in etomidate potentiation (G2 + E1 *vs*. G2 + K + E1; *n* = 7; *p* < 0.01; paired *t* test), but had no effect on etomidate activation (E2 *vs*. E2 + K; *n* = 6; *p* > 0.05; paired *t* test). Data are normalised to the 10 mM GABA responses, and are presented as mean ± SEM.

At 3 μM, etomidate enhanced the GABA EC_3_ responses efficaciously (53 ± 2.6% of maximal GABA responses; *n* = 7; Fig 5). The addition of 300 μM kavain resulted in a modest, but significant reduction in potentiation (45 ± 3.1% of maximal GABA responses; *n* = 7; *p* < 0.01; paired *t* test). At 30 μM, etomidate directly elicited current responses approximately 24 ± 4.2% of the maximal GABA amplitude (*n* = 6). The agonist effect was not significantly altered in the presence of 300 μM kavain (27 ± 2.8%; *n* = 6; *p* > 0.05; paired *t* test).

#### Propofol

**Fig 6:**
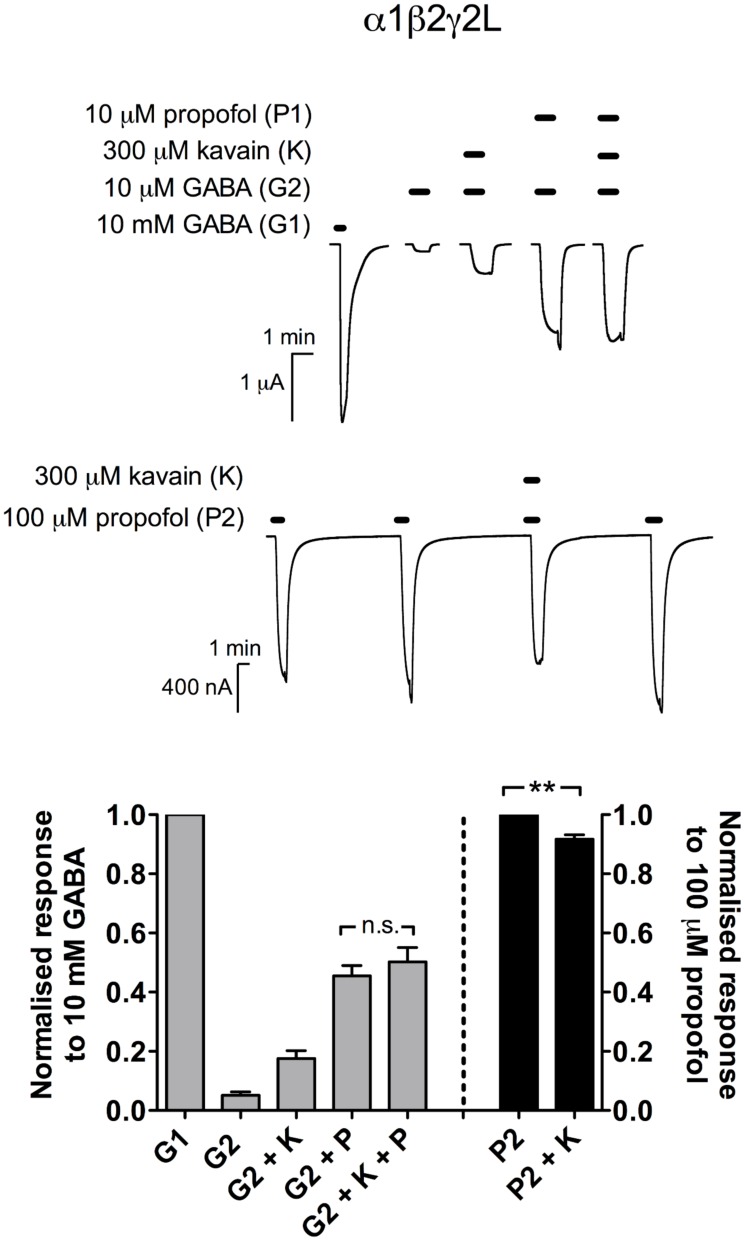
Kavain did not affect propofol potentiation, but modestly reduced propofol activation at α1β2γ2L GABA_A_Rs. *Top*, Representative traces of current responses to 10 mM GABA (control); 10 μM GABA; 10 μM GABA and 300 μM kavain; 10 μM GABA and 10 μM propofol; 10 μM GABA, 300 μM kavain and 10 μM propofol. *Middle*, Continuous traces demonstrating two consecutive applications of control (100 μM propofol) followed by the co-application of 300 μM kavain with control; and control. *Bottom*, Receptor modulation produced by propofol alone (G2 + P) was not significantly different from the combination of kavain and propofol (G2 + K + P; *n* = 5; *p* > 0.05; paired *t* test). The agonist effect of propofol (P2) was significantly reduced in the presence of kavain (P2 + K; *n* = 5; *p* < 0.01; paired *t* test). Data are presented as mean ± SEM.

At 10 μM, propofol enhanced the GABA EC_3_ responses efficaciously (45 ± 3.5% of the maximal GABA responses; *n* = 5; Fig 6). The addition of 300 μM kavain resulted in a slight, but insignificant enhancement in propofol potentiation (50 ± 4.8%; *p* > 0.05; paired *t* test). Following exposure to 100 μM propofol, GABA-elicited current responses were highly variable, resulting in inconsistent measurement. Thus, the agonist effect of propofol was investigated in the absence of GABA. We found that when combined, the currents elicited by 300 μM kavain and 100 μM propofol were smaller than the application of propofol alone (91 ± 1.5% of the maximal propofol responses; *n* = 5; *p* < 0.01; paired *t* test; Fig 6).

### Kavain-mediated modulation at GABA_A_Rs containing α1M236W, β2M286W and β3N265M mutations

Next, we assessed the possibility of kavain interacting with the transmembrane β+α‒ anaesthetic binding sites. To this end, we introduced the α1M236W, β2M286W and β3N265M point mutations which perturb etomidate and propofol actions, and investigated the impact of these mutations on kavain activity at GABA_A_Rs.

#### α1M236Wβ2γ2L

The co-expression of α1M236W with β2 and γ2L subunits resulted in receptors with higher GABA potency (EC_50_ = 59 μM; *n* = 7) than wild-type α1β2γ2L GABA_A_Rs (EC_50_ = 136 μM; *p* < 0.01 for logEC_50_ comparisons; unpaired *t* test; Table 1). The point mutation significantly attenuated the modulatory effects of 3 μM etomidate (*p* < 0.0001; *n* = 5; unpaired *t* test; data not shown) and 10 μM propofol (*p* < 0.01; *n* = 5; unpaired *t* test) on GABA EC_3_ (1 μM) responses. In contrast, the agonist effects of 30 μM etomidate and 100 μM propofol appeared to be greater, but were not significantly different from wild-type receptors (*p* > 0.05; *n* = 5 for both etomidate and propofol; unpaired *t* test). Overall, these functional characteristics are consistent with previous studies [34].

**Fig 7:**
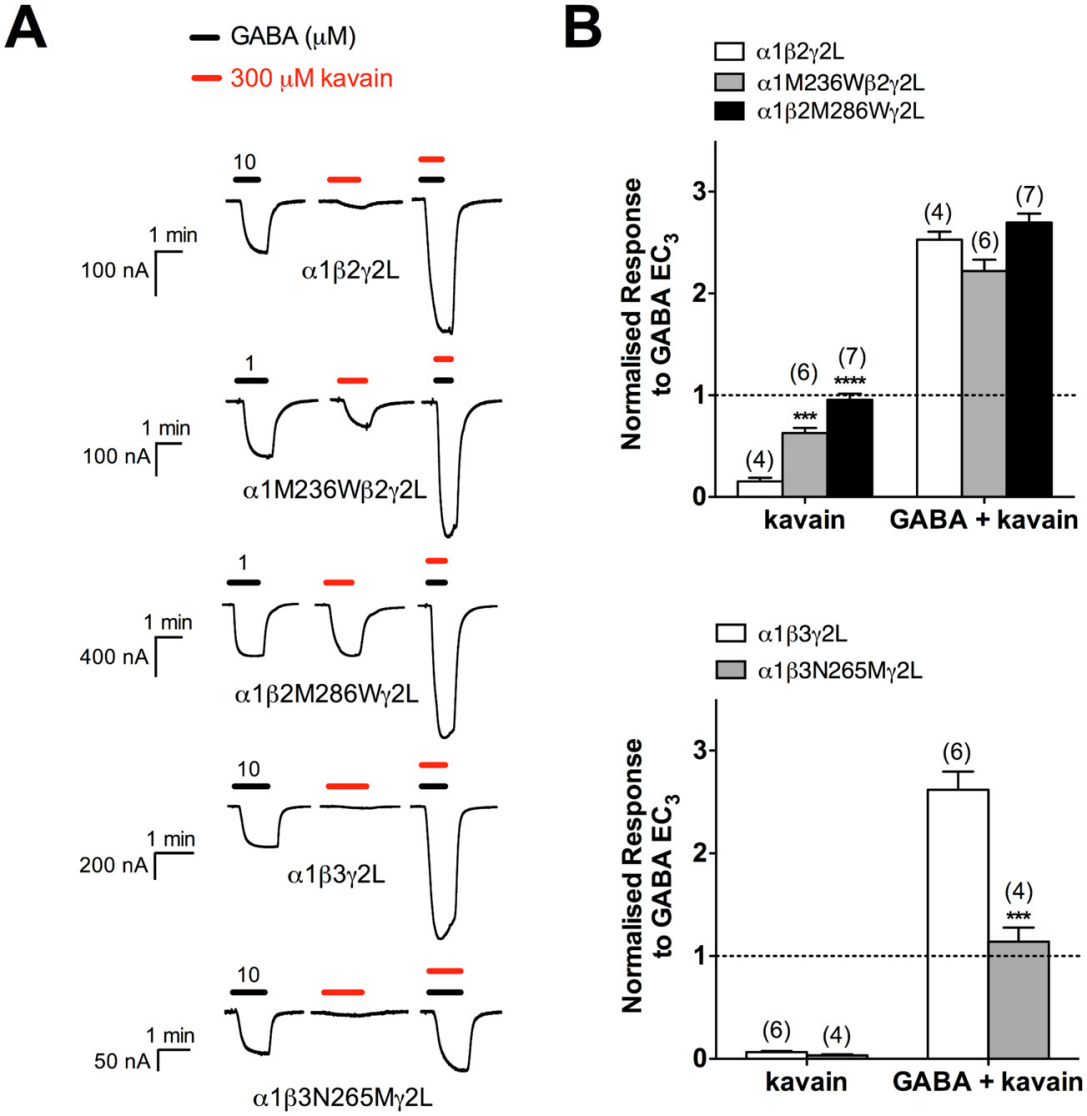
The pronounced effect of β3N265M point mutation on etomidate and propofol sensitivity. (A) Representative traces demonstrating the modulatory effect of 3 μM etomidate and 10 μM propofol on GABA EC_3_ (*left*) and the agonist effect of 30 μM etomidate and 100 μM propofol relative to 10 mM GABA (*right*) at α1β3γ2L and α1β3N265Mγ2L GABA_A_Rs. (B) The modulatory and agonist effects of etomidate and propofol were markedly diminished at α1β3N265Mγ2L GABA_A_Rs. Data are normalised to current responses elicited by 10 mM GABA, and are presented as mean ± SEM. *** *p* < 0.001; **** *p* < 0.0001; unpaired *t* test (mutant *vs*. wild-type). Numbers above bars indicate number of experiments. GABA + ETO: GABA EC_3_ + 3 μM etomidate; ETO: 30 μM etomidate; GABA + PRO: GABA EC_3_ + 10 μM propofol; PRO: 100 μM propofol.

Next, we investigated the effect of kavain alone and in combination with GABA EC_3_ at α1M236Wβ2γ2L GABA_A_Rs. At 300 μM, kavain elicited current responses approximately 63 ± 5.2% of the GABA EC_3_ responses (*n* = 6), which was significantly greater than that observed at wild-type receptors (15 ± 3.6%; *n* = 4; *p* < 0.001; unpaired *t* test; Fig 7). The degree of potentiation produced by the co-application of GABA EC_3_ and 300 μM kavain, however, was not significantly different between α1β2γ2L (250 ± 7.8% of GABA EC_3_ responses; *n* = 4) and α1M236Wβ2γ2L (220 ± 11%; *n* = 6) GABA_A_Rs (*p* > 0.05; unpaired *t* test). Note: kavain-elicited current amplitudes were not deducted from the measurement of kavain-induced GABA potentiation.

#### α1β2M286Wγ2L

Receptors expressed from α1, β2M286W and γ2L subunits displayed enhanced GABA sensitivity (EC_50_ = 25 μM; *n* = 5; *p* < 0.0001 for logEC_50_ comparisons *vs*. α1β2γ2L; unpaired *t* test; Table 1). In agreement with previous studies, etomidate sensitivity was abolished with the introduction of this point mutation, which is evident from the absence of modulatory and agonist effects (data now shown) [34]. Receptor modulation produced by 10 μM propofol in the presence of GABA EC_3_ (1 μM) was also significantly diminished (*p* < 0.001; unpaired *t* test). However, the direct activation caused by 100 μM propofol was unaffected (*p* > 0.05; unpaired *t* test). Similar propofol profile at α1β2M286Wγ2S receptors has been reported [35]. Like α1M236Wβ2γ2L, α1β2M286Wγ2L receptors showed increased efficacy in response to 300 μM kavain alone (96 ± 5.8% of the GABA EC_3_ responses; *p* < 0.0001; unpaired *t* test; Fig 7). The modulatory effect of kavain on GABA EC_3_-elicited current responses, however, was not significantly different from α1β2γ2L GABA_A_Rs (270 ± 8.7% of GABA EC_3_; *p* > 0.05; unpaired *t* test; Fig 7).

#### α1β3N265Mγ2L

The asparagine-to-methionine mutation at position 265 of the β2/3 subunits is known to profoundly weaken anaesthetic sensitivity with minimal effect on GABA activity [36, 37]. In agreement with previous studies, we found that GABA potencies at both α1β3γ2L and α1β3N265Mγ2L GABA_A_Rs were not significantly different (*p* > 0.05 for logEC_50_ comparisons; unpaired *t* test; Table 1). Etomidate did not modulate (at 3 μM) nor activate (at 30 μM) the point-mutated receptors (data not shown). Pronounced attenuation of propofol activity was also observed. In contrast to the α1M236W and β2M286W mutations, 300 μM kavain had negligible action by itself at α1β3N265Mγ2L receptors (3.3 ± 1.0% of GABA EC_3_ responses). However, kavain modulation of GABA EC_3_ responses was significantly reduced from 260 ± 18% at α1β3γ2L to 110 ± 14% at α1β3N265Mγ2L receptors (*p* < 0.001; unpaired *t* test; Fig 7).

## Discussion

In this study, we characterised the functional effects of kavain at 9 different human GABA_A_R subtypes expressed in *Xenopus* oocytes, and found no subtype dependence in kavain potentiation (Fig 2C). The concentration-response relationships of kavain at all receptor subtypes investigated were steep, with no plateau obtained at the solubility limit of the compound. Thus, we could not accurately determine the potency of kavain. The inference on the lack of subtype selectivity was based on the modulation efficacy of currents elicited by a low concentration of GABA by the highest soluble concentration of kavain (300 μM). Our results demonstrated that kavain potentiation did not require the α1 or γ2L subunit, as both β2γ2L and α1β2 GABA_A_Rs were modulated to similar extent as α1β2γ2L GABA_A_Rs. Furthermore, varying the α (α1, 2, 3 or 5) and β (β1, 2 or 3) subunit isoforms in αβγ2L receptors, or replacing the γ2L subunit with δ (α4β2δ) did not result in significant differences in kavain potentiation.

However, the modulatory actions of kavain at α1β2γ2L and α4β2δ GABA_A_Rs are functionally different (Fig 3). Kavain modulated α1β2γ2L GABA_A_Rs at GABA concentrations below the EC_45_, but failed to potentiate peak GABA currents. In contrast, the GABA concentration-response curve of α4β2δ GABA_A_Rs was significantly shifted upwards in the presence of kavain. The functional preference of kavain for α4β2δ GABA_A_Rs can be explained by the partial-agonist, weak-intrinsic-efficacy profile of GABA at this receptor subtype that renders it more sensitive to kavain modulation. Similar differential enhancement effects that arise from the distinct GABA efficacies at synaptic αβγ and extrasynaptic αβδ receptors have been described for other allosteric ligands such as etomidate [38], propofol [39], pentobarbital [40], and neurosteroids [41]. While our findings do not conclusively prove that GABA_A_Rs are the *in vivo* substrate for kavain, it is tempting to speculate that kavain may have a larger physiological impact on extrasynaptic than synaptic GABA_A_Rs. This conjecture is substantiated by previous studies that found more prominent kavain or kavalactones actions in brain regions such as the hippocampus [21, 42], in which tonic GABAergic conductances mediated via δ-containing GABA_A_Rs have been detected [43–45]. Future electrophysiological studies comparing kavain activity in neurons from wild-type and α4- or δ-knockout mice will be necessary to confirm this speculation.

The low affinity of kavain detected in our study is in agreement with most studies conducted in the past using kava extracts or pure kavalactones [21, 22, 46]. The high-micromolar concentrations required to observe the pharmacological actions of kavalactones *in vitro* raise the critical question of whether the concentrations used in these studies are physiologically relevant. Studies conducted in mice showed that 100 mg/kg of kavain alone caused a rapid surge in brain concentration (up to 100 μM) within minutes after *i*.*p*. injection [22, 47]. A higher brain concentration of kavain was found (120 μM) in the presence of other kavalactones (44 mg kavain in 120 mg/kg kavalactone mixture; *i*.*p*.), clearly demonstrating pharmacokinetic synergism [22, 47]. Davies *et al*. (1992) predicted higher brain concentrations (300 μM) with the administration of larger doses (300 mg/kg) of kavain [22]. Thus, kavain concentrations used in our study appear to correlate well with the concentrations needed to induce anxiolysis and hypnosis in mice [27, 48]. Unfortunately, the relevance of this concentration range in humans cannot be established as no data is currently available [49].

The possibility of kavalactones interacting non-specifically with lipid membrane of neurons to exert their psychoactive effects has been raised [21, 22]. However, our mutational studies revealed a near-complete loss of kavain potentiation at α1β3N265Mγ2L GABA_A_Rs (Fig 7). The extensively studied β3N265M point mutation is known to selectively abolish the *in vitro* and *in vivo* sensitivity of anaesthetics such as etomidate, propofol, pentobarbital and volatile agents [50]. As such, this finding argues strongly against the lipid hypothesis, and supports a direct kavain-GABA_A_R interaction. We also investigated the impact of mutating two homologous methionine residues (α1M236 and β2M286) that line the transmembrane β+α‒ anaesthetic binding sites. Substituting the methionine residues with tryptophan decreases etomidate and propofol sensitivity, an effect which has been correlated with increased side-chain volume of tryptophan sterically hindering anaesthetic binding [34]. Unlike β3N265M, the α1M236W and β2M286W point mutations did not negatively affect the activity of kavain (Fig 7), suggesting that either the binding determinants of kavain are different from etomidate and propofol, or kavain does not bind to the transmembrane β+α‒ interfaces. The enhanced agonist efficacy of kavain observed can be explained by the higher open probability of these spontaneously-gated mutant receptors [34].

We infer, based on the preliminary mutational evidence, that kavain binds to the transmembrane anaesthetic sites. Two observations support this notion: (1) The β3N265M point mutation impairs the sensitivity of anaesthetics agents that bind to homologous transmembrane cavities, but not benzodiazepines and neurosteroids which target binding sites of dissimilar locations. (2) Kavain displays remarkably similar pharmacological profile to the volatile anaesthetics which exhibit low affinity (EC_50_ > 200 μM) and steep concentration-response relationships, besides lacking subtype selectivity at GABA_A_Rs [51, 52]. However, given the absence of subtype specificity in kavain modulation at GABA_A_Rs, the existence of multiple binding sites for kavain is highly probable. Future studies are warranted to provide a more comprehensive picture of the kavain binding site(s).

As part of our efforts in characterising the interaction of kavain and other GABA_A_R modulators on a receptor level, we found that kavain action was not blocked by flumazenil (Fig 4). This finding corroborates previous studies that failed to detect any significant interaction of kavalactones with the high-affinity benzodiazepine site [19, 21–23, 46]. In addition, kavain and diazepam modulated GABA_A_Rs in a less-than-additive manner, contrary to the speculated synergistic (supra-additive) interaction [32]. This finding is surprising as two positive modulators that bind to distinct, independent binding sites typically give rise to additive potentiation when combined, as exemplified by sevoflurane and propofol [53], and diazepam and valerenic acid [54]. As flumazenil insensitivity of kavain action indicates a non-benzodiazepine mechanism for the kavalactone, the lack of additivity may be due to (1) the putative kavain binding site(s) being allosterically coupled to the classical benzodiazepine site in an inhibitory fashion, resulting in less-than-additive joint enhancement, or (2) kavain affecting a common transduction pathway that reduces the enhancement efficacy by diazepam at GABA_A_Rs.

## Conclusions

Despite the long history of kava consumption and the wealth of clinical evidence in favour of the efficacy of kavalactones in treating anxiety, there is a severe gap in our understanding of the molecular target(s) and the mechanism(s) of action of these psychoactive compounds. In this study, we have demonstrated that the enhancement of GABA_A_R function by kavain occurs in a subtype non-selective and flumazenil-insensitive manner. Furthermore, we provide the first experimental evidence that supports a direct interaction of a major kavalactone with GABA_A_Rs *in vitro*. It is expected that the identification of specific GABA_A_R-enhancing kavalactones in the future will give impetus to the development and refinement of these kava-derived chemicals as novel anxiolytics.
